# Developing Tools for Identifying Employer and Employee Satisfaction of Nursing New Graduates in China

**DOI:** 10.1155/2014/384649

**Published:** 2014-07-03

**Authors:** Yuying Fan, Qiujie Li, Shufen Yang, Ying Guo, Libin Yang, Shibin Zhao

**Affiliations:** ^1^The Second Affiliated Hospital & College of Nursing, Harbin Medical University, Xuefu Road 246, Harbin, Heilongjiang Province 150086, China; ^2^The Second Affiliated Hospital, Harbin Medical University, Harbin, Heilongjiang 150086, China; ^3^Department of Medicine Education and Research, Harbin Medical University, No. 246 Xuefu Road, Harbin, Heilongjiang 150086, China

## Abstract

*Purpose.* Researchers developed evaluation tools measuring employment relevant satisfaction for nursing new graduates. The evaluation tools were designed to be relevant to nursing managers who make employment decisions and nursing new graduates who were just employed. *Methods.* In-depth interviews and an expert panel were established to review the activities that evaluate the employee and employer satisfaction of nursing new graduates. Based on individual interviews and literature review, evaluation items were selected. A two-round Delphi study was then conducted from September 2008 to May 2009 with a panel of experts from a range of nursing colleges in China. *Results.* The response rate was 100% and Kendall's W was 0.73 in the second round of Delphi study. After two rounds of Delphi surveys, a list of 5 employee satisfaction items and 4 employer satisfaction items was identified for nursing new graduates. *Conclusions.* The findings of this study identified a different but multidimensional set of factors for employment relevant satisfaction, which confirmed the importance of certain fundamental aspects of practice. We developed the evaluation tools to assess the employer and employee satisfaction of nursing new graduates, which provided a database for further study.

## 1. Introduction

China is currently focusing on the employment of university graduates. Challenges arise in identifying factors that are relevant to employment satisfaction. Employment brings income, which has become the primary concern for new employees. However, other factors are important, such as personal development, dignity, self-identification, and self-actualization.

The employment relevant satisfaction could be defined as a level to reflect the job situation and inherent features to which university graduates are satisfied. This concept is a subjective and, as illustrated above, multidimensional concept. Its broadest definition includes the objective characteristics related to employer satisfaction (both specific to the job and general relating to the wider labor market such as health and safety standards), characteristics of the worker, the match between worker and employment characteristics, and the worker's subjective evaluation (employee satisfaction) of the employment characteristics [1].

To be examined from the microcosmic field, the employment relevant satisfaction for university graduates covers all of the features of the jobs which university graduates get, such as job income, working hours, job environment, the graduates' satisfaction, the stability of job, the harmony of labor relationship, career development prospects, and social insurance. To be explored from macroscope, the employment relevant satisfaction is affected by the government and social economy situations to a great extent [2].

In China, nursing education at university level began in the 1980s [3]. Graduates are awarded a Bachelor of Medicine degree and become registered nurses (RNs) after passing a national RN examination. Over 300 nursing colleges provide nursing baccalaureate programs in Mainland China [4]. It is estimated that as many as 5,000 nursing students graduate annually from these programs. Nurses with baccalaureate degrees assumed significant roles during this period.

In the 1990s, only a few nurses worked in clinical settings after obtaining a Bachelor's degree, whereas a large number of nurses worked in managerial and faculty capacities [5]. The current employment of nurses has changed a lot, and most nursing baccalaureate graduates opt to practice after graduation [4].

Based on a longitudinal survey on nursing graduate students in a university in China, 34.3% of the graduates expressed optimism about their job prospects, 22.9% prioritized money, and 18.9% considered the personal development opportunity supported by employers as an important factor [6]. The authors also reported that 79.8% of nursing graduates regarded larger hospitals with favour, and 96.8% preferred to work in private hospitals that are in excellent conditions [6]. Another study with a sample size of over 300 nursing baccalaureate graduates in Hebei Province in China reported that 69% of nursing graduates moved to big cities because of rapid economic development [7].

A survey conducted in New Zealand on job candidates revealed the following 10 most common reasons for employment: (1) lack of career progression, (2) seeking new challenges, (3) salary, (4) lack of training or development opportunities, (5) poor management, (6) too much stress, (7) too much traveling time, (8) seeking to specialize in a particular field, (9) poor work/life balance, and (10) office politics [8]. The primary reason for employment was the “lack of career progression or not.”

In Canada, an Internet survey with a 50% response rate revealed that in choosing a future career, students employed in Federal Public Service for the summer ranked the following five factors as the most important from a list of 15: (1) interesting work (62.9%), (2) competitive salary (51.7%), (3) personal development (41.6%), (4) balanced work and personal lives (29.6%), and (5) long-term job security (22.2%) [9].

Chinese researchers conducted a survey on the employment of graduates from universities in Beijing. They investigated the graduates from 1995 to 2005 and presented three aspects to evaluate employment satisfaction, namely, (1) salary, (2) nature of labor contract, and (3) training [10].

Another survey conducted in China explored the degree of employer satisfaction. Ke suggested using the following four factors to evaluate employer satisfaction: (1) knowledge level, (2) moral base and professional values, (3) core competencies, and (4) health situation of employees [11].

The following useful conclusions were derived from the analysis of the literature.

1. Many studies focused on employment status but not on employment satisfaction.
2. The satisfaction of both employers and employees was considered when employment relevant satisfaction was evaluated.

In the current study, China has no nationally recognized specialty standards for assessing the employment relevant satisfaction of nursing new graduates (ERSNNG). Greater attention should be paid to employment satisfaction since satisfaction could affect the living quality tremendously. Our study aimed to develop tools to assess employment relevant satisfaction of nursing baccalaureate graduates in China.

## 2. Ethical Approval

This study was conducted with the approval of the Ethics Committee of The Second Affiliated Hospital of Harbin Medical University. The main ethical considerations for this study included obtaining informed consent and ensuring anonymity as much as possible. The research team secured all relevant approvals before commencing the process.

## 3. Methods

### 3.1. Student and Graduate Sample

Factors which nursing graduates considered as important ones were determined before measuring employment relevant satisfaction. The characteristics of job that workers consider as the most important will determine the meaningfulness of the job for them, and the satisfaction they derive from it. Thirteen nursing baccalaureate students (who were also intern nursing students seeking jobs) and 17 registered nurses working in hospitals who had already graduated from nursing colleges were selected for face-to-face interviews. The interviews aimed to determine the job expectations of those students and nurses.

### 3.2. In-Depth Individual Interviews

Each interviewee was briefed about the research and written informed consent was obtained from all participants. The interview began with two open-ended questions: (1) please describe the ideal job that you expect to have and (2) what features do you think a good job should have?

All interviews were recorded with a digital voice recorder. This gave researchers the ability to review the interviews and generate detailed insights. The qualitative data analytical strategy involved the following six steps: (1) listening and reading all records carefully, (2) extracting important and recurring statements, (3) encoding the statements, (4) writing down the detailed description, (5) identifying similar statements, and (6) synthesizing theme statements to extract the factors from the responses.

After the individual interviews, 84 items of which the ratio was over 50% remained to form the questionnaire.

### 3.3. Expert Sample

Purposive stratified sampling was used to establish the Delphi panel. Experts in a particular field were selected to obtain their expertise on special issues based on their knowledge and practical engagement [12]. The criteria for the recruitment of experts included positions above the associate-professor level, engagement in nursing education for at least 10 years, and membership in local nursing associations. The panel members were required to be knowledgeable about and familiar with the employment status of nursing graduates, as well as credible within their profession.

### 3.4. Delphi Method

The Delphi technique was used to systematically review the items in the evaluation tool or identifying the employment satisfaction of nursing baccalaureate graduates. In the first round, the panel (*n* = 22) was sent a questionnaire containing a list of 84 employment satisfaction items collected from the interviews. Experts considered the importance of each item.

The experts were also asked whether there were additional items not mentioned in the tool. They could add items to further improve the tool. After analyzing the responses, the researchers revised the tool and formulated the second-round questionnaire sending it to the same expert panel. The panel was invited to rate the items again, and those experts who disagreed with the aggregate group judgment on any particular attribute or attributes were asked to briefly explain their disagreement.

The agreement criteria for rating each item used in this study were based on descriptive summary statistics, and the assumption that the scale upon which panel members expressed their opinions was measured at the interval level. The mean, standard deviation (SD), and variation coefficient for the rating of each item were calculated in each round of the survey.

## 4. Measurements

Panel members were asked to rate the importance of each individual attribute on a five-point Likert scale. The participants were also asked to rate the feasibility for each attribute on a five-point Likert scale (1 = not at all feasible, 2 = moderately feasible, 3 = feasible, 4 = very feasible, and 5 = always feasible).

Kendall's W was used to assess the reliability of the ERSNNG. Kendall's W is an important coefficient that reveals a significant level of concordance between assessors in the evaluation process. This coefficient is a measure of the agreement among several judges who are assessing a given set of objects [13]. Each rank order is represented by the set of all pairs of objects, and the value of 1 or 0 is assigned to this pair when its order corresponds or does not correspond to the manner in which the two objects are ordered. The higher the coefficient gets, the more consistent the analysis is.

The weight of each item was analyzed using the percentage method. The results included the overall mean weight rating by participants.

## 5. Results

**Figure 1:**
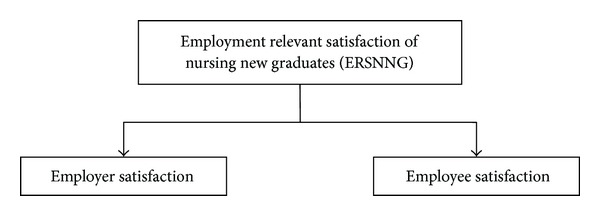
Model for employment relevant satisfaction of nursing new graduates.

*About ERSNNG.* After two rounds using the Delphi method, the results of this study indicated that the ERSNNG could be assessed by two major constructs, namely, employer satisfaction and employee satisfaction (Figure 1). Employer satisfaction could be evaluated by theoretical knowledge, moral and personal values, core nursing competencies, and health situation of graduates. Employee satisfaction could be judged by employment situation, job stability, job remuneration, personal development, and work-life balance. The weight of each item was then calculated (Table 1).

**Figure 2:**
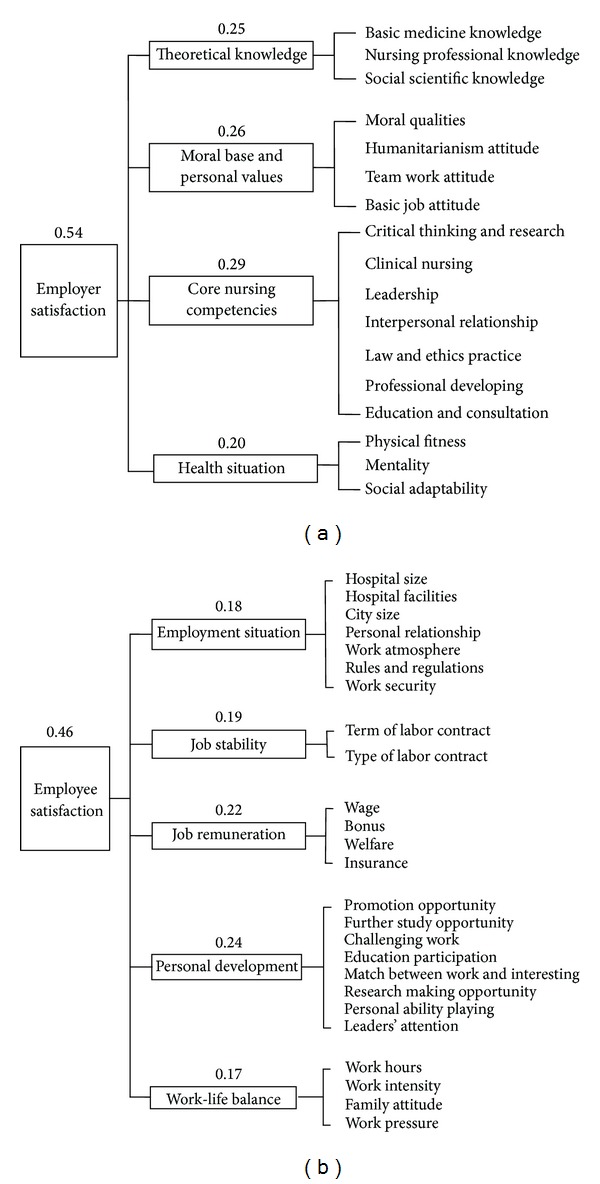
(a) The tool for employer satisfaction. (b) The tool for employee satisfaction.

The results indicated that the importance of employer satisfaction was 0.54 and that of employee satisfaction was 0.46 (Figure 2). Employer satisfaction comprised four dimensions, namely, theoretical knowledge, moral base and personal values, core nursing competencies, and health situation. The results implied that the employers paid more attention to the item of core nursing competencies (29.38%).

Employee satisfaction comprised five dimensions, namely, employment situation, job stability, job remuneration, personal development, and work-life balance.

## 6. Analysis

### 6.1. Baccalaureate Students and Graduate Nurses

The mean age of the participants was 22.7 years (SD = 1.16). The 17 registered nurses had work experience from 1 to 3 years.

### 6.2. Experts

The expert panel comprised 22 experts in nursing education, nursing management, and community nursing. Among the experts, 16 (72.73%) had Master's degrees and two had Ph.Ds., with the remainder having completed certificate, Bachelor's degree, or postgraduate certificate qualifications. Moreover, 18 (81.82%) experts worked for over 20 years, and six (27.27%) experts served in the nursing field for over 30 years (Table 2). The same expert panel was used for all Delphi rounds, with three persons discontinuing after the first round (response rate was 88%). In the second round of inquiry, 22 questionnaires were sent out and the return rate was 100%. A response rate of over 70% was suggested by Sumsion [14] for each round to maintain the rigour of the Delphi technique.

### 6.3. Kendall's W Coefficient

Kendall's W in this study was 0.56 in the first round and 0.73 in the second round (Table 3).

## 7. Discussion

The employment relevant satisfaction of nursing new graduates could be judged from two major aspects, namely, employer satisfaction and employee satisfaction. Our findings indicated that when employers recruited nurses, they focused on employee knowledge level, moral base and professional values, core nursing competencies, and health situation. This result was similar to the Chinese research [11]. These assessment items are applicable to all health and social care staff; they focus on components valued by managers.

Experts valued the core nursing competencies as the most important factor among the four factors (Table 1). Evidence also suggested that the core nursing competencies were identified as the most important factor which reflected an orientation towards a person-centered approach to care [15]. In general, highly satisfying jobs are also the most productive ones and require higher levels of skills. Quality nursing care is also affected by the competencies of nurses. Numerous studies have articulated that individual competencies are related to employment satisfaction. Hence, acquiring better competencies could help nursing new graduates obtain higher employment satisfaction. The weight of professional development ability ranked first among all core nursing competencies. Nursing graduates are expected to identify clinical nursing problems, independently collect clinical data, appropriately use research findings, resolve problems, and enhance nursing professional development.

A number of researchers used the item of employee satisfaction to assess employment status [16]. Job satisfaction could be used to predict behaviour [17]. In our research, five items contributed to employee satisfaction (in no particular order), namely, employment situation, job stability, job remuneration, personal development, and work-life balance.

Our results suggested that personal development and career progress were the most valued item among Chinese nursing graduates (24.06%, Table 1). This factor motivated Chinese nursing graduates most to stay in a job or search for a new job. This result was similar to the study conducted in New Zealand [8]. Professional development involves all types of facilitated learning opportunities, ranging from college degrees to formal coursework, conferences, and informal learning opportunities. Furthermore, professional development enhances and maintains employment satisfaction.

Other important values included job remuneration and job stability. The employment situation and work-life balance also mattered but less than the other factors did. Taken together, these results indicated that personal growth and intrinsic job characteristics were more valued than the other factors.

Evaluating employee satisfaction involved determining whether employees were happy at work. Our study indicated that money was not the only measurement for employee satisfaction. One of the best means of improving employee satisfaction was to increase personal development and career progress for nurses. Personal development, together with bonuses, could also seriously affect the job satisfaction of nurses.

Employee satisfaction and employer satisfaction captured a range of characteristics and considered the personal values and subjective opinions of nursing graduates. These two factors also captured the characteristics that are difficult to measure, such as job interest. Identifying which of the two factors is more important is difficult, as both factors contribute to the concept of employment relevant satisfaction.

Using the Delphi method has several limitations, many of which are related to its scientific credibility. First, agreement is lacking regarding the size of the panel or any recommendations concerning sampling techniques. According to an appraisal of several studies, the range of panel size seemed to arbitrarily vary. Critique of the Delphi listed 13 published studies in health applications in which the size of the panel varied from 10 to 1,685 (range 1,585). Moreover, the follow-up response rate in numerous studies decreased in inverse proportion to the panel size. Therefore, the validity of the results is subject to response bias.

With regard to the panel composition, few studies specified the criteria for selection. The notion that panel members were experts seems to be implicit, as they were selected rather than fulfilling any specific standards. Another unresolved question pertains to the definition of an expert, and if expert opinion is distinguishable from that of anyone else. Defining an expert must be arbitrary. We selected 25 experts in the first round of inquiry, but three experts declined to participate in this research. Therefore, only 22 experts were included in our research.

This study has numerous implications for agencies employing nurses. Once a nurse is hired it is important that she stay on at the agency. The key to retaining and using talent is how to encourage staff members to develop their creativity and allow for professional growth. The value of highly qualified and educated nurses has been increasingly recognized by hospitals. Employee satisfaction has become an important method of enhancing the performance of hospitals.

Further possible use of this tool is to obtain data on the employment satisfaction of nursing baccalaureate graduates. Two kinds of questionnaires would be required to use this model. One questionnaire should be distributed to employers and the other to nursing graduates. The questionnaire scores would provide important data for nursing leaders and planners.

## 8. Conclusion

Information was gathered to assess the employment satisfaction of nursing baccalaureate graduates, with a database for further study. After defining employment satisfaction, the next phase will involve determining interventions to increase employment satisfaction. In addition, more research is needed to assess the reliability and validity of the instrument.

In sum, the findings of this study identified a different but multidimensional set of factors for employment relevant satisfaction, which confirmed the importance of certain fundamental aspects of practice. The study provided important data on strategies for reforming and developing nursing education through the evaluation of the employment satisfaction of nursing graduates.

## Figures and Tables

**Table 1:** Items of tools for employment relevant satisfaction.

Items	Importance (%)	Standard deviation
*Employer satisfaction *	54.39	9.47
**Theoretical knowledge** (basic medical knowledge, nursing professional knowledge, and social scientific knowledge)	25.63	5.9
**Moral base and personal values** (moral qualities, humanitarianism attitude, team work attitude, and basic job attitude)	26.56	1.49
**Core nursing competencies** (critical thinking and research, clinical nursing, leadership, interpersonal relationship, law and ethics practice, professional development, education, and consultation)	29.38	7.93
**Health situation** (physical fitness, mentality, and social adaptability)	19.82	4.81

*Employee satisfaction *	45.61	7.24
**Employment situation** (hospital size, hospital facilities, city size, interpersonal relationship, work atmosphere, rules and regulations, and work security)	18.11	8.46
**Job stability** (term of labor contract, type of labor contract)	19.13	6.55
**Job remuneration** (wage, bonus, welfare, and insurance)	22.08	8.21
**Personal development** (promotion opportunity, further study opportunity, challenging work, education participation, work and interesting match, research making opportunity, personal ability playing, and leaders' attention)	24.06	9.64
**Work-life balance** (work hours, work intensity, family attitude, and work pressure)	16.62	6.05

**Table 2:** Description of statistical analysis of experts (*n* = 22).

Characteristic	*n* (%)
Ages	
30–39 y	2 (9.09)
40–49 y	16 (72.73)
50–59 y	4 (18.18)
Title	
Associate professor	15 (68.18)
Professor	7 (31.82)
Working years	
10–19 years	4 (18.18)
20–29 years	12 (54.55)
30–39 years	6 (27.27)
Working and studying field	
Nursing education	9 (40.91)
Nursing management	11 (50.00)
Community nursing	2 (9.09)

**Table 3:** Kendall's *W* and Chi-squire test of experts.

	The first round	The second round
Kendall coefficient	0.56	0.73
*χ*_*R*_^2^	301.27	752.95
*P*	<0.01	<0.01
